# A novel Mcl-1 inhibitor synergizes with venetoclax to induce apoptosis in cancer cells

**DOI:** 10.1186/s10020-022-00565-7

**Published:** 2023-01-19

**Authors:** Tianming Zhao, Qiang He, Shurong Xie, Huien Zhan, Cheng Jiang, Shengbin Lin, Fangshu Liu, Cong Wang, Guo Chen, Hui Zeng

**Affiliations:** 1 Department of Hematology, The First Affiliated Hospital of Jinan University, Guangzhou, 510630 China; 2 Department of Medical Biochemistry and Molecular Biology, School of Medicine, Jinan University, Guangzhou, 510632 China; 3 Jiang Su Key Laboratory of Drug Design and Optimization, Department of Medicinal Chemistry, China Pharmaceutical University, Nanjing, 210009 China; 4 School of Biopharmacy, China Pharmaceutical University, Nanjing, 211198 China

**Keywords:** Mcl-1 inhibitor, Bcl-2, Venetoclax, Apoptosis, AML

## Abstract

**Background:**

Evading apoptosis by overexpression of anti-apoptotic Bcl-2 family proteins is a hallmark of cancer cells and the Bcl-2 selective inhibitor venetoclax is widely used in the treatment of hematologic malignancies. Mcl-1, another anti-apoptotic Bcl-2 family member, is recognized as the primary cause of resistance to venetoclax treatment. However, there is currently no Mcl-1 inhibitor approved for clinical use.

**Methods:**

Paired parental and Mcl-1 knockout H1299 cells were used to screen and identify a small molecule named MI-238. Immunoprecipitation (IP) and flow cytometry assay were performed to analyze the activation of pro-apoptotic protein Bak. Annexin V staining and western blot analysis of cleaved caspase 3 were employed to measure the cell apoptosis. Mouse xenograft AML model using luciferase-expressing Molm13 cells was employed to evaluate in vivo therapeutic efficacy. Bone marrow samples from newly diagnosed AML patients were collected to evaluate the therapeutic potency.

**Results:**

Here, we show that MI-238, a novel and specific Mcl-1 inhibitor, can disrupt the association of Mcl-1 with BH3-only pro-apoptotic proteins, selectively leading to apoptosis in Mcl-1 proficient cells. Moreover, MI-238 treatment also potently induces apoptosis in acute myeloid leukemia (AML) cells. Notably, the combined treatment of MI-238 with venetoclax exhibited strong synergistic anti-cancer effects in AML cells in vitro, MOLM-13 xenografts mouse model and AML patient samples.

**Conclusions:**

This study identified a novel and selective Mcl-1 inhibitor MI-238 and demonstrated that the development of MI-238 provides a novel strategy to improve the outcome of venetoclax therapy in AML.

**Supplementary Information:**

The online version contains supplementary material available at 10.1186/s10020-022-00565-7.

## Introduction

Apoptosis, one of the programmed cell death, occurs normally during development and aging, and it plays critical roles in maintaining tissue homeostasis (Carneiro and El-Deiry 2020). Two major types of conserved signaling pathways, including intrinsic and extrinsic pathway have been established to execute apoptosis (Fulda and Debatin 2006). Intrinsic apoptosis is associated with mitochondria outer membrane permeabilization (MOMP), which subsequently causes the release of cytochrome C from mitochondria into cytosol to activate a cascade of caspases cysteine proteases (Chen et al. 2019; Hamacher-Brady et al. 2014). While, extrinsic apoptosis is regulated by death receptors binding to its ligands, such as TNFR1/TNF (Sayers 2011; Karstedt et al. 2017). The intrinsic mitochondria apoptosis is controlled by Bcl-2 family proteins, which share structural homology in one to four conserved regions named Bcl-2 homology (BH) domain (Warren et al. 2019). In response to apoptotic stimuli, pro-apoptotic Bcl-2 proteins, such as Bax and Bak, oligomerize at the mitochondrial outer membrane and trigger MOMP (Busche et al. 2021; Holzerland et al. 2020; Huang et al. 2019). Meanwhile, anti-apoptotic Bcl-2 proteins, such as Bcl-2, Mcl-1 and Bcl-xL, reside in the mitochondrial outer membrane and prevent pro-apoptotic protein mediated oligomerization and MOMP (Warren et al. 2019; Wei et al. 2020).

The major anti-apoptotic Bcl-2 family members including Bcl-2, Mcl-1 and Bcl-xL have been observed to be overexpressed in various cancer cells, which confer cancer cell resistance to apoptosis (Warren et al. 2019). Therefore, targeting these anti-apoptotic proteins to induce apoptosis has been utilized as a useful strategy to treat and prevent cancers (Bajpai et al. 2020; Han et al. 2015). The anti-apoptotic Bcl-2 family members possess structural similarity to bind on BH3 domain of pro-apoptotic proteins, and this hydrophobic surface binding pocket within anti-apoptotic Bcl-2 proteins is named BH3 binding pocket, which is required for its anti-apoptotic function (Woo et al. 2003). Synthetic peptides that fit into this pocket have been shown to induce apoptosis in vitro and in vivo (LaBelle et al. 2012). Through structural-based drug design, small-molecule BH3 mimetics such as ABT-199, ABT-737 and ABT-263, which could bind to the hydrophobic pocket, have been developed (Lagares et al. 2017; Pan et al. 2014; Ritschka et al. 2020). These small-molecule BH3 mimetics induce apoptosis in various cancer cells and possess potent anti-tumor efficacy (Pan et al. 2014). Through a long period of clinical trials, ABT-199 (trade name venetoclax) was finally approved as monotherapy or combination therapy for the treatment of hematologic malignancies including chronic lymphocytic leukemia (CLL) and acute myeloid leukemia (AML) (Ramsey et al. 2018).

However, venetoclax selectively binds to Bcl-2 and has limited efficacy against cancer cells that depends on other anti-apoptotic proteins for survival such as Mcl-1 (Souers et al. 2013). Treatment of venetoclax has been reported to increase the binding of Mcl-1 to pro-apoptotic proteins, such as Bax, Bim and Bak (Luedtke et al. 2017). Therefore, it is well-established that Mcl-1 plays critical roles in venetoclax resistance, and combination treatment of venetoclax with Mcl-1 inhibitors induces synergistic anti-tumor activity and eradicates venetoclax-resistant cancers (Ramsey et al. 2018; Luedtke et al. 2017). Therefore, specific Mcl-1 inhibitor could be used not only as apoptosis inducer but also in venetoclax combination therapy. Although several Mcl-1 inhibitors have been developed and exhibited promising anti-tumor efficacy in pre-clinical leukemia or solid tumor animal models (Ramsey et al. 2018; Dengler et al. 2020; Kotschy et al. 2016), there is still a lack of Mcl-1 inhibitors approved for clinical use. In the present study, we show that a novel small molecule named MI-238 could bind to Mcl-1 and selectively induced apoptosis in Mcl-1 proficient cells, but not in Mcl-1 deficient cells. The combined treatment with venetoclax and MI-238 induced synergistic anti-tumor effects in AML cells in vitro, xenograft mouse model and patient samples.

## Materials and methods

### Cell lines, plasmids and antibodies

Parental and Mcl-1 knockout (KO) H1299 cells were maintained in RPMI 1640 medium supplemented with 10% FBS as our previously described (Chen et al. 2018). Wild type (WT) and Mcl-1 KO MEF (mouse embryonic fibroblast) cells were cultured in DMEM medium supplemented with 10% FBS as previously described (Chen et al. 2018). Anti-Mcl-1 (#94296), anti-cleaved caspase 3 (#9661) and anti-Bim (#2933) were purchased from Cell Signaling Technology (MA, USA). Anti-Bcl-2 (sc-7382), anti-Bax (sc-7480), anti-PARP1 (sc-8007) and β-Actin (sc-8432) were obtained from Santa Cruz Biotechnology (CA, USA). Anti-Cytochrome C (ab133504), Anti-Bcl-xL (ab32370) and Anti-Bak (ab32371) were purchased from Abcam (Cambridge, MA).

### Knockout of Mcl-1 cells

Mcl-1 KO cells were established as previously described (Chen et al. 2018).

### Immunoprecipitation and GST pull-down assay

Cells were lysated in ice-cold EBC buffer (0.5% NP-40, 50 mM Tris–HCl, pH 7.6, 120 mM NaCl, 5 mM CaCl_2_, 5 mM Mgcl_2_ and 1 mM β-mercaptoethanol) with protease inhibitor cocktail (TargetMol, China) by sonication. After centrifuge, the cell lysates were incubated with anti-Mcl-1, anti-Bcl-2, or anti-Bcl-xL antibody and Protein A/G-agarose beads (Santa Cruz, CA) overnight at 4 ℃ with rotation. After washing, beads were boiled in 30 µl SDS-PAGE loading buffer for 6 min and subjected to SDS-PAGE and analyzed by Western blotting. For GST pull-down assay, GST-fused Mcl-1 proteins were incubated with glutathione sepharose 4B beads (GE healthcare) in TBS buffer (50 mM Tris–Cl, pH 7.5, 150 mM NaCl) with protease inhibitor cocktail at 4 ℃ for 4 h. After washing, the beads coated with GST-Mcl-1 proteins were incubated with recombinant Bak protein in TBS buffer in presence of increasing concentrations of MI-238 at 4 ℃ overnight. After washing, the samples were subjected to SDS-PAGE and analyzed by Western blotting.

### Annexin-V staining assay

Cells were treated with MI-238 or venetoclax for 48 h before apoptosis analysis. The percentage of apoptotic cells were measure using annexin-V apoptosis detection kit (BD Biosciences, NJ, USA) according to the manufacturer’s instruction. Briefly, 1 × 10^6^ of drug treated cells were incubated with annexin-V-FITC in binding buffer for 15 min in dark at room temperature. Then, 50 μg/ml of propidium iodide (PI) was added before analyzing by flow cytometry.

### Flow cytometry assay of Bak activation

For the detection of conformational changes of Bak, 2 × 10^6^ of Molm13 cells were fixed with 2% paraformaldehyde (Sangon Biotech) for 30 min on ice, followed by permeabilization with 0.5% triton X-100 (Sangon Biotech) for 30 min at room temperature and blocking with 5% goat serum for 30 min at room temperature. Cells were then labeled for 30 min with 1 mg/mL of antibodies against the active form of Bak (clone G317-2; BD Pharmingen). After incubation with FITC-conjugated anti-mouse secondary antibody (Cell Signaling Technology), cells were analyzed by flow cytometry.

### Immunohistochemistry (IHC)

Mouse bone marrow was harvested for Immunohistochemistry to identify AML burden. Briefly, Mice were anesthetized by inhaling isoflurane. The stripped tibia or femur was fixed in 4% paraformaldehyde, and decalcified in EDTA regent for 2 weeks, embedded in paraffin. Longitudinal sections (4 µm) of tibia or femur were prepared. After antigen retrieval, the slices were incubated in 3% (V/V) H_2_O_2_ at room temperature for 25 min to block endogenous peroxidase activity. Nonspecific staining was further blocked with BSA at room temperature for 30 min. The sections were incubated with anti-hCD45 (Cell Signaling Technology) antibody at 4 ℃ overnight. The corresponding secondary antibody incubated the tissue for 50 min at room temperature. Then, the peroxidase reaction was observed with DAB peroxidase substrate. After counterstaining with hematoxylin, the slides were dehydrated, mounted, and visualized with Leica light microscope (Leica, Wetzlar, Germany).

### Patient samples

Bone marrow samples from newly diagnosed AML patients were used to evaluate the anti-leukemia response of MI-238. AML patients and donor volunteers involved in this study signed a consent form. Bone marrow mononuclear cells were isolated by Ficoll gradient centrifugation. Then, primary AML cells were cultured in α-MEM medium supplemented with 20% FBS, 1% penicillin/streptomycin, and 10 ng/ml recombinant human cytokine, including SCF, TPO, FLT-3 ligand, IL-3 and IL-6. All cytokines were purchased from Peprotech (NJ, USA). The primary AML cells were cultured with different concentrations of MI-238 or in combination with venetoclax for 48 h before apoptosis analysis by flow cytometry.

### Xenograft model

Cell line-derived xenograft (CDX) was implemented to confirm the anti-leukemia effect of MI-238 in vivo as previously described (Pan et al. 2014). Female NCG (NOD/ShiLtJGpt-Prkdc^em26Cd52^Il2rg^em26Cd22^/Gpt) mice, 8–10 weeks old, were inoculated with 7 × 10^5^ (in 200 μl PBS) luciferase-expressing Molm13 cells through the tail vein. After the AML mouse model was successfully constructed, MI-238 (70 mg/kg) or vehicle was intraperitoneally injected, and venetoclax (50 mg/kg) or vehicle was administered by daily gavage for 2 weeks. On days10, 17 and 24, AML burden was determined by fluorescence imaging. On day 24, the proportion of hCD45/hCD33 (Biolegend, 368504 and 366618) cells in peripheral blood was detected by flow cytometry. The above experimental protocol was approved by the ethics committee of Jinan University.

### Statistical analysis

All data were presented as mean ± standard deviation (SD) from at least three independent replicates. Statistical comparisons of two samples were performed using two-tailed student’s t-test and *P* < 0.05 was considered as statistical significance. Kaplan–Meier method was performed to analyze differences in the animal survival.

## Results

### MI-238 is a novel and potent Mcl-1 inhibitor

**Fig. 1:**
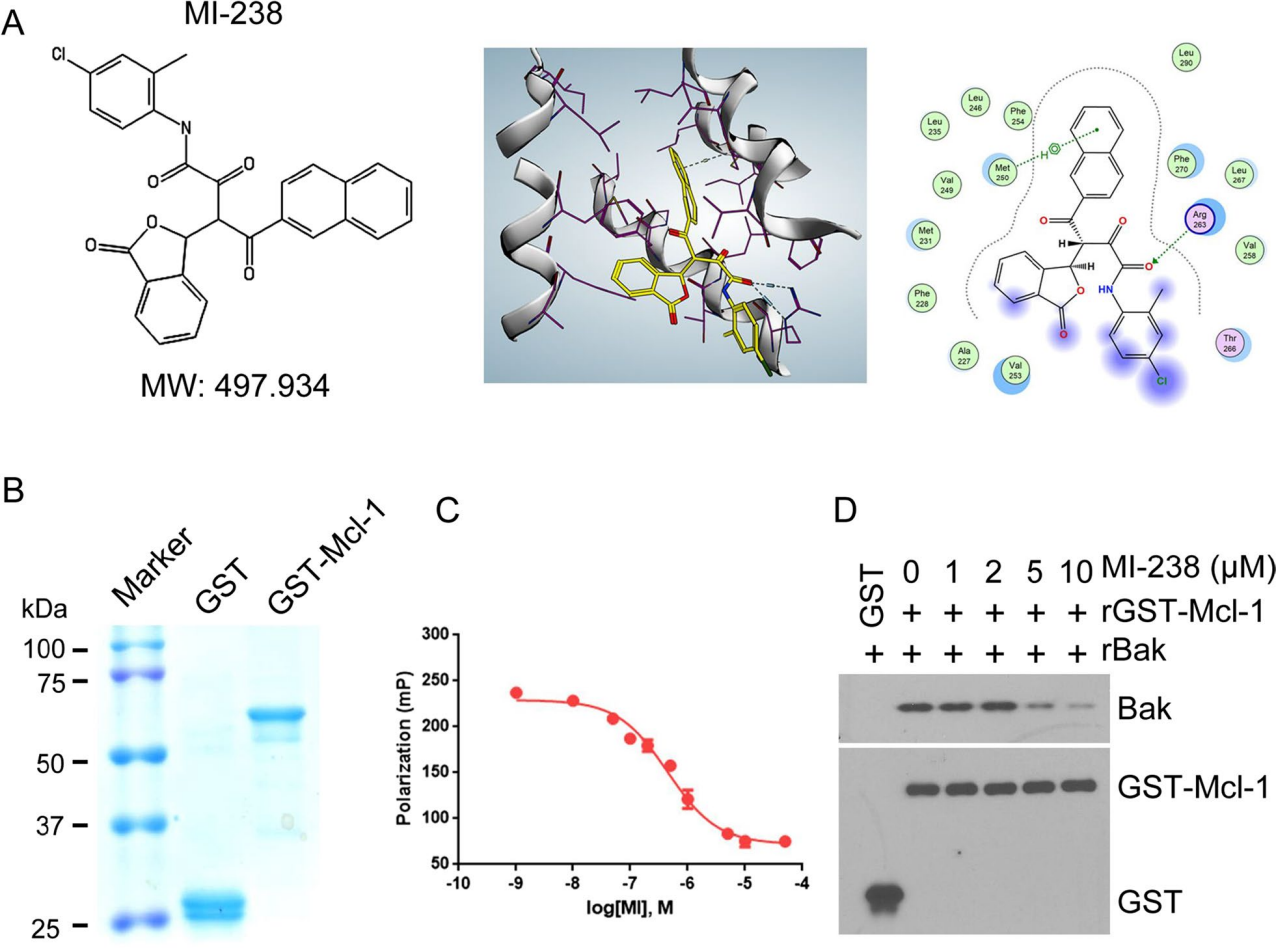
MI-238 targets Mcl-1 and disrupts Mcl-1/Bak association. **A** Chemical information and structure modeling of MI-238 in the BH3-binding pocket of Mcl-1 (PDB ID: 4HW3). **B** Purification of recombinant GST or GST-Mcl-1 proteins. **C** Fluorescence polarization (FP) assay was performed to measure the binding of MI-238 to recombinant GST-Mcl-1 protein. Data are represented as mean ± SD, n = 3. **D** GST pull down assay was conducted to analyze the association between recombinant GST-Mcl-1 (rGST-Mcl-1) and recombinant Bak (rBak) in presence of increasing concentrations of MI-238

As an important pro-survival protein, Mcl-1 is over-expressed in various types of cancer. However, no specific Mcl-1 inhibitor is currently available for clinical use. Through cytotoxicity screening using the paired H1299 parental and Mcl-1 knockout (KO) cells, we discovered a small molecule that selectively inhibited the viability of H1299 parental cells, but not Mcl-1 KO cells (Additional file 1: Fig. S1), and we named this compound MI-238 (Fig. 1A). MI-238 was docked into Mcl-1 BH3 binding groove and proximal to the BH1 domain of Mcl-1 (Fig. 1A). To directly measure MI-238/Mcl-1 binding, we prepared GST tagged Mcl-1 recombinant protein (Fig. 1B). MI-238 exhibited a Ki of 0.45 ± 0.05 μM to human Mcl-1 protein in FP (fluorescence polarization) assay (Fig. 1C). Mcl-1 exerts its pro-survival function through binding with BH3-only pro-apoptotic proteins, such as Bak. To test whether MI-238 binding to Mcl-1 could disrupt Mcl-1/Bak association, purified Mcl-1 and Bak proteins were incubated in presence of increasing concentrations of MI-238. As shown in Fig. 1D, MI-238 disrupted Bak/Mcl-1 interaction in a dose-dependent manner in vitro. Collectively, these results demonstrated that MI-238 directly binds to Mcl-1 and inhibits Mcl-1 anti-apoptotic function.

### MI-238 selectively induces apoptosis in Mcl-1 proficient cells

**Fig. 2:**
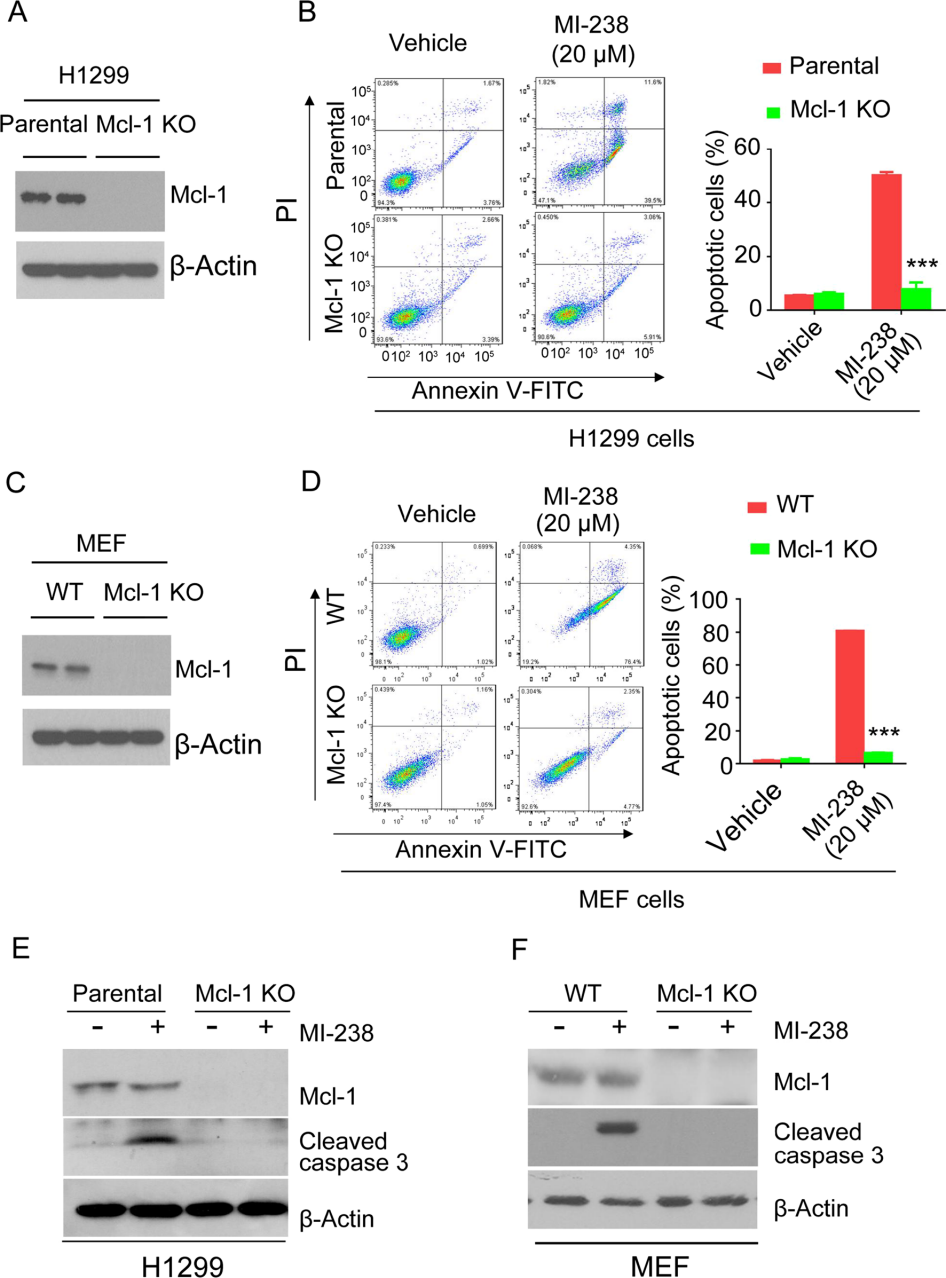
MI-238 selectively induces apoptosis in Mcl-1 proficient cells. **A** Western blotting analysis of indicated protein expressions in cell lysate derived from H1299 parental or Mcl-1 knockout (KO) cells. **B** H1299 parental or Mcl-1 KO cells were treated with or without 20 μM MI-238 for 48 h, and the cell apoptosis were analyzed by annexin V staining. **C** Western blot analysis as above were performed in MEF wild-type (WT) or Mcl-1 KO cells. **D** Apoptosis analysis as above were performed in MEF wild-type (WT) or Mcl-1 KO cells. **E**, **F** Cell apoptosis were analyzed by caspase 3 cleavage in H1299 (**E**) or MEF (**F**). Data are represented as mean ± SD from three independent replicates, ***P < 0.001 by two-tailed t-test

To measure whether MI-238 induced apoptosis depends on Mcl-1, we generated H1299 Mcl-1 KO cells (Fig. 2A, C) (Chen et al. 2018). Knockout of Mcl-1 in H1299 and MEF cells did not significantly affect the expression Bcl-2 protein. However, BH3-only proteins including Bak, Bim and Bax were shifted to Bcl-2 and Bcl-xL in Mcl-1 deficient cells, which indicated Mcl-1 KO cells relied more on Bcl-2 and Bcl-xL for survival (Additional file 1: Fig. S2). The annexin V apoptosis assay revealed that 20 μM of MI-238 treatment induced apoptosis in 50.1 ± 1.1% of parental H1299 cells, but intriguingly, 20 μM MI-238 failed to induce apoptosis in Mcl-1 deficient H1299 cells (Fig. 2B). Cleavage of caspase 3 initiates apoptotic DNA fragmentation and is recognized as an apoptosis hallmark (Carneiro and El-Deiry 2020). Consistent with the annexin V assay, MI-238 treatment caused caspase 3 cleavage in H1299 parental cells, but not in Mcl-1 KO cells (Fig. 2E). Similarly, we found that MI-238 treatment also induced apoptosis in wild type (WT) mouse embryonic fibroblast (MEF) cells, but not in Mcl-1 KO MEF cells (Fig. 2D, F). These results demonstrated that MI-238 induced apoptosis is dependent on proficient Mcl-1.

### MI-238 effectively kills AML cells

**Fig. 3:**
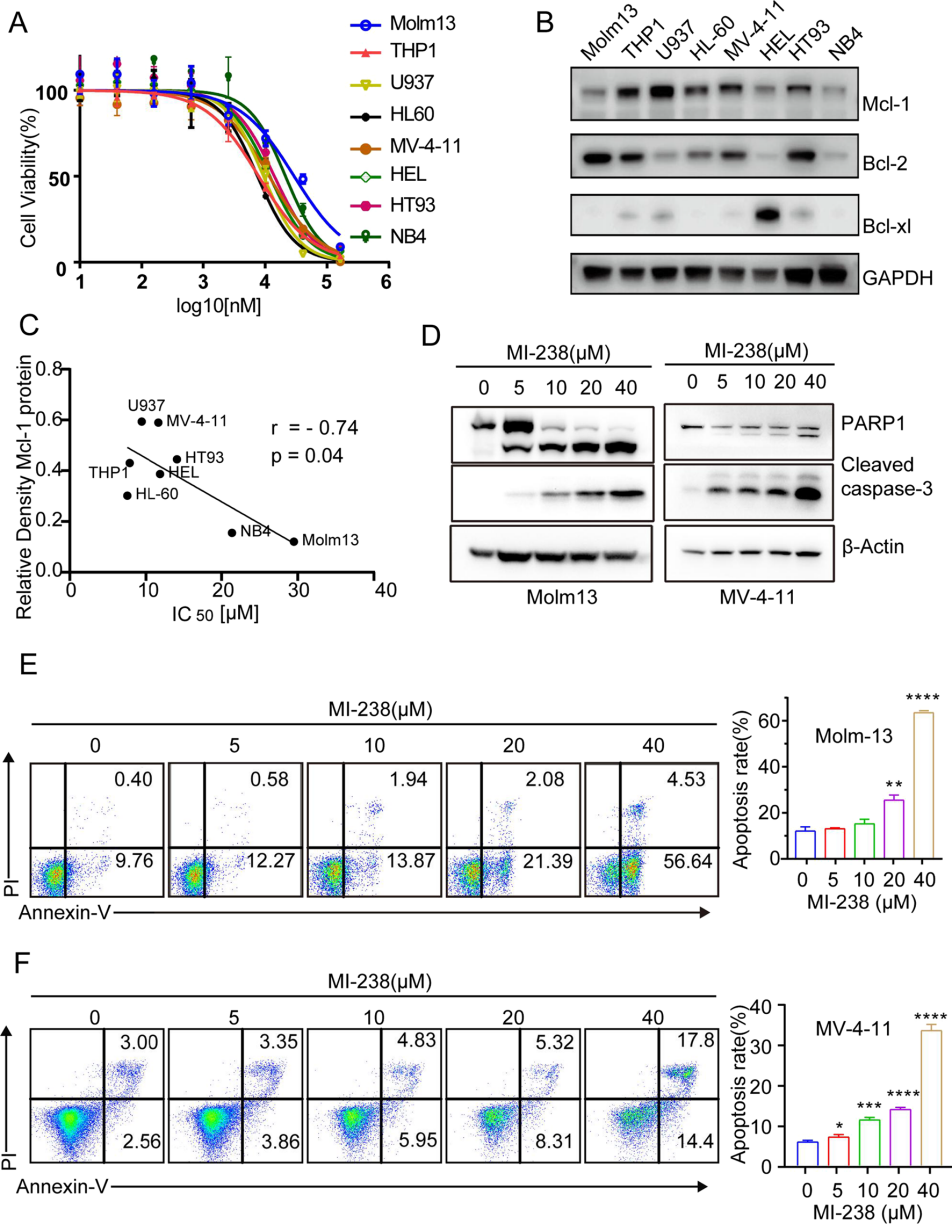
MI-238 effectively induces apoptosis in AML cells. **A** The cell viability of indicated 8 AML cell lines in presence of increasing concentrations of MI-238 were analyzed by cell counting kit-8 (CCK8) assay and the half-maximal inhibitory concentration (IC_50_) IC_50_ were determined. **B** Western blot analysis of the expression of Mcl-1, Bcl-2 and Bcl-xL in AML cell lines. **C** Correlation of Mcl-1 protein level and the IC_50_ of MI-238 were determined. **D**, **E**, **F** Molm13 and MV-4-11 cells were treated with increasing concentrations of MI-238 for 48 h, and the cell apoptosis was analyzed by western blot analysis of PARP1 and caspase 3 cleavage (**D**) and flow cytometry of annexin V staining (**E**, **F**). Data are represented as mean ± SD from three independent replicates, *P < 0.05, **P < 0.01,  ***P < 0.001, ****P < 0.0001 by two-tailed t-test

Induction of apoptosis through targeting Bcl-2 anti-apoptotic proteins is an effective therapeutic strategy for hematologic malignancies, and Bcl-2 inhibitor venetoclax is widely used to treat AML and CLL (Ramsey et al. 2018). To test the therapeutic efficacy of MI-238 in AML cells, we treated a variety of AML cells with increasing concentrations of MI-238 and found that the IC_50_ of MI-238 against AML is around 5–30 μM (Fig. 3A). In addition, the IC_50_ of MI-238 was inversely proportional to the Mcl-1 expression levels among AML cells (Fig. 3B, C, Additional file 1: Fig. S3). We then examined the PARP1 and caspase 3 cleavage, the well-known apoptosis markers in Molm13 and MV-4–11 cells after MI-238 treatment (Carneiro and El-Deiry 2020). As shown in Fig. 3D, MI-238 induced PARP1 and caspase 3 cleavage in a dose-dependent manner, which indicated MI-238 potently induces apoptosis in AML cells. We further employed annexin V staining to measure the apoptosis frequency in Molm13 and MV-4-11 cells after MI-238 treatment, and found that 40 μM of MI-238 caused 60 ± 0.4% and 35 ± 1.5% apoptotic cell death in Molm13 and MV-4-11 cells respectively (Fig. 3E, F). Taken together, these results demonstrated that MI-238 effectively induces apoptosis in AML cells.

### MI-238 treatment induces activation of BH3-only proteins

**Fig. 4:**
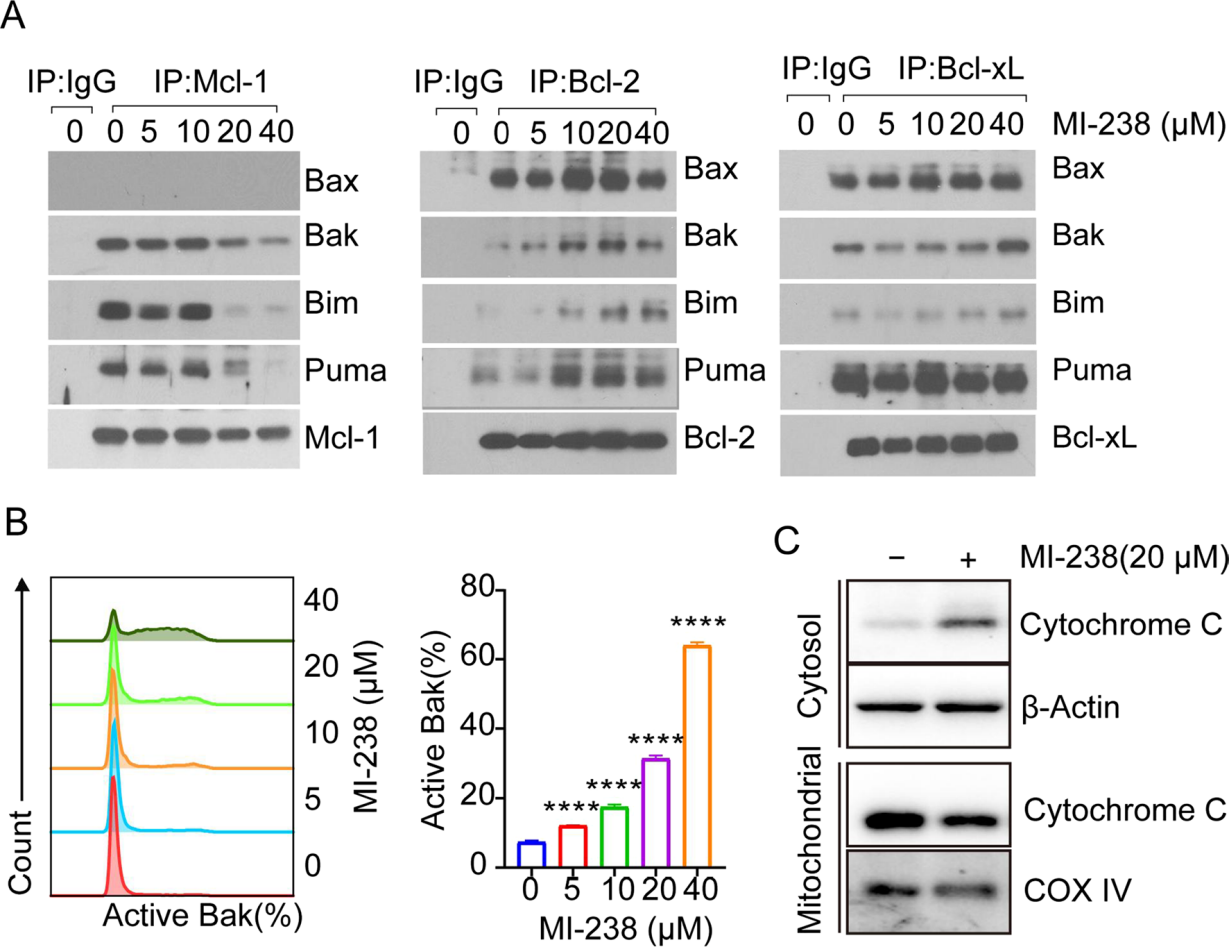
MI-238 disrupts the association of Mcl-1 with BH3-only proteins. **A** Molm13 cells were treated with indicated concentrations with MI-238 for 24 h, followed by Immunoprecipitation (IP) assay using anti-Mcl-1, anti-Bcl2 and anti-Bcl-xL antibodies and western blot analysis with indicated BH3-only proteins. **B** Molm13 cells were treated with indicated concentrations of MI-238, followed by flow cytometry analysis of Bak activation using the antibody (clone: G317-2) specifically recognized activated Bak. Data are presented as mean ± SD from three independent replicates, ****P < 0.0001 by two-tailed t-test. **C** Molm13 cells were treated with or without 20 μM MI-238 for 24 h. Then, subcellular fractionation was subsequently performed and the cytochrome C level in mitochondria and cytosol were analyzed by western blot

Mcl-1 inhibits apoptosis by sequestering pro-apoptotic BH3-only proteins, such as Bax, Bak, Bim and Puma (Kotschy et al. 2016). To check whether MI-238 treatment could release BH3-only proteins from Mcl-1, we performed an immunoprecipitation (IP) assay using antibodies against anti-apoptotic proteins including Mcl-1, Bcl-2 and Bcl-xL, and the result showed that Mcl-1 mainly binds to Bak, Bim, and Puma, but not Bax in Molm13 cells (Fig. 4A). Meanwhile, treatment of MI-238 could disrupt Mcl-1 association with BH3-only pro-apoptotic proteins including Bak, Bim and Puma (Fig. 4A). Whereas, MI-238 failed to interrupt the binding of BH3-only proteins to Bcl-2 and Bcl-xL (Fig. 4A), suggesting MI-238 treatment specifically inhibits Mcl-1, but not Bcl-2 and Bcl-xL. Bak release from Mcl-1 causes its conformation change and homo-oligomerization to initiate apoptosis. We then employed flow cytometry analysis of Bak activation after MI-238 treatment by staining with activation-specific antibody. Consistent with the IP assay, MI-238 treatment induced Bak activation in Molm13 cells in a dose-dependent manner (Fig. 4B).

Activation of BH3-only proteins results in the release of cytochrome C from the mitochondria into the cytosol, which in turn triggers apoptosis (Kluck et al. 1997). We then performed cell fractionation analysis to examine cytochrome C levels in mitochondrial and cytosol after MI-238 treatment. As shown in Fig. 4C, we observed a significant decrease of mitochondrial cytochrome C level and increase cytosol cytochrome C after MI-238 treatment, indicating MI-238 could induce cytochrome C translocation from mitochondrial to cytosol.

### MI-238 synergizes with venetoclax to induce apoptosis in AML cells

**Fig. 5:**
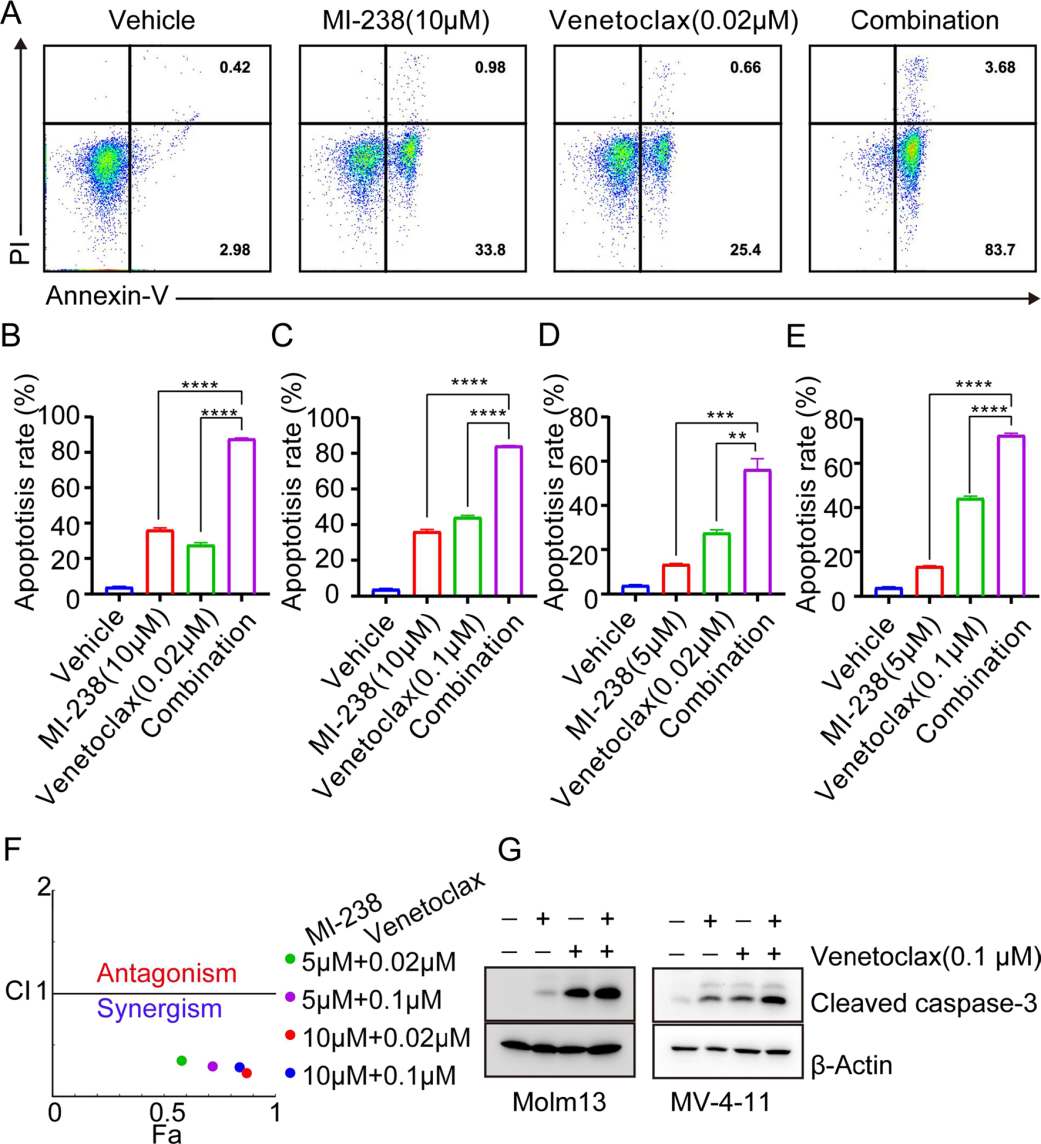
MI-238 synergizes with venetoclax to induce apoptosis in AML cells. **A**–**E** Molm13 cells were treated with indicated concentrations of MI-238, venetoclax, or their combination for 48 h, followed by apoptosis assay by annexin V staining. Data are represented as mean ± SD from three independent replicates, **P < 0.01, ***P < 0.001, and ****P < 0.0001 by two-tailed t-test. **F** The combination index (CI) was calculated by CompuSyn software. **G** Molm13 and MV-4-11 cells were treated as indicated, and the caspase 3 cleavage was analyzed by western blot

Given that Mcl-1 is the primary venetoclax-resistant protein (Ramsey et al. 2018), we then tested whether MI-238 could sensitize AML cells to venetoclax treatment. As shown in Fig. 5A, B, 10 μM of MI-238 induced 34.8 ± 1.2% apoptosis, 0.02 μM of venetoclax caused 26.1 ± 1.3% apoptosis in Molm13 cells, while, their combination induced 87.4 ± 0.3% apoptosis. Besides, synergistic effects of MI-238 and venetoclax on apoptosis induction in Molm13 cells have also been detected in different combinations (10 μM + 0.1 μM, 5 μM + 0.02 μM, and 5 μM + 0.1 μM). (Fig. 5A–F, and Additional file 1: Fig. S4). Consistent with the annexin V staining assay, the combination treatment of MI-238 and venetoclax induced greater caspase 3 cleavage compared with the treatment of MI-238 only or venetoclax only (Fig. 5G). In addition, MI-238 and venetoclax combination induced significantly greater activation of Bak compared with MI-238 or venetoclax treatment alone (Additional file 1: Fig. S5). These results indicate that MI-238 could sensitize AML cells to venetoclax treatment and combination of MI-238 and venetoclax induces synergistic anti-tumor effects.

### MI-238 and venetoclax have a synergistic effect in AML xenografts

**Fig. 6:**
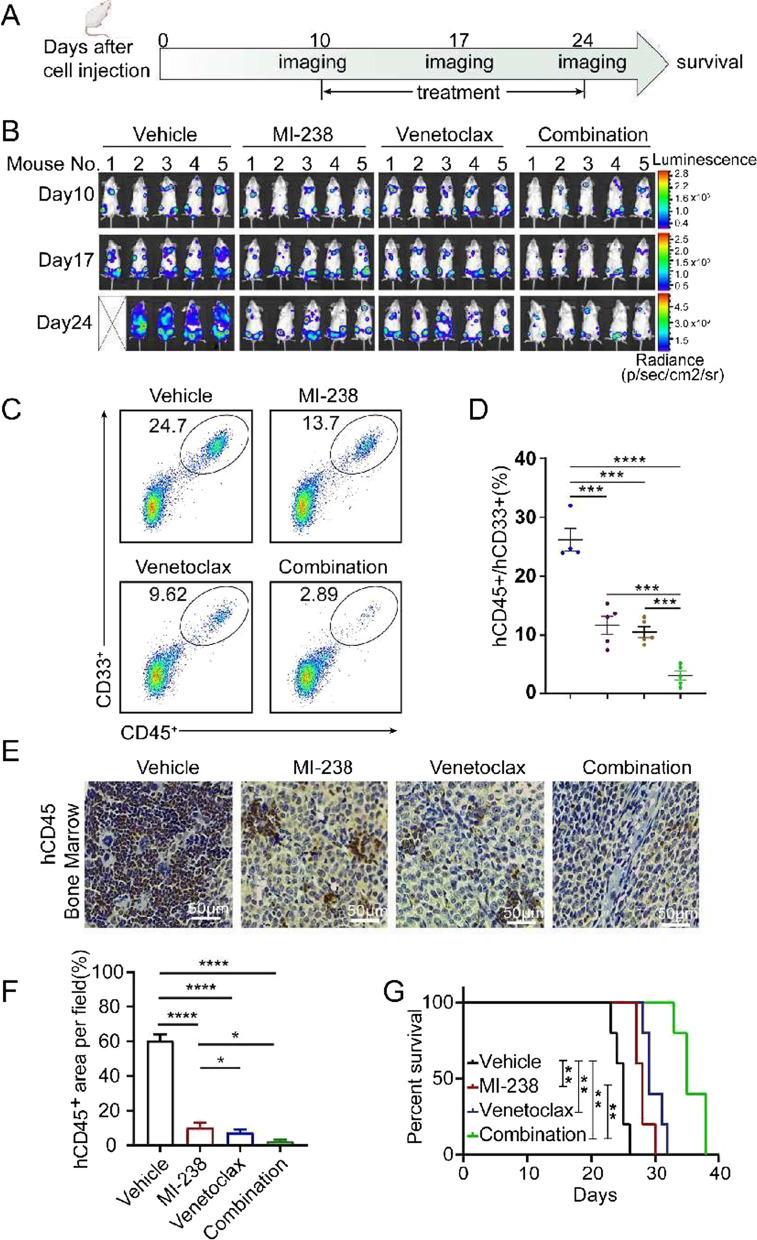
The combination of MI-238 and venetoclax potently inhibited the development of AML in murine model. **A** Schematic diagram of the experimental design showing the timeline for the treatment and imaging. **B** Representative bioluminescent images of the Molm13 tumor burden in mice treated with vehicle, MI-238 (70 mg/kg), venetoclax (50 mg/kg) or their combination. **C**, **D** The percentage of human CD45 (hCD45) and human CD33 (hCD33) positive cells were analyzed by flow cytometry to measure the Molm13 tumor burden. The representative flow cytometry plots (**C**) and quantification of hCD45 and hCD33 double positive (hCD45^+^/hCD33^+^) cells (**D**) were shown. Data are represented as mean ± SD from three independent replicates. ***P < 0.001 and ****P < 0.0001 by two-tailed t-test. **E**, **F** Immunochemistry (IHC) analysis of hCD45 expression in bone marrow from experimental mice. Representative staining (**E**) and quantification (**F**) were shown. Data represent mean ± SD from three independent replicates, *P < 0.05 and ****P < 0.0001 by two-tailed t-test. **G** Kaplan–Meier analysis showed MI-238 in combination with venetoclax resulted in a survival benefit in Molm13 AML xenograft mice. **P < 0.01 by log-rank (Mantel–Cox) test (n = 5)

To evaluate the therapeutic efficacy of MI-238 and venetoclax in vivo, mice were intravenously (i.v) injected with Molm13 cells stably expressing luciferase (Molm13-Luc) to generate Molm13 AML xenograft model. We started treatment at 10 days after cell implantation, and monitored the cancer progression once a week by the bioluminescence imaging (Fig. 6A). At the beginning of the treatment (10 days after Molm13 implantation), we clearly detected luciferase signal in all mice, that is proportional to amounts of leukemic cells (Fig. 6B). Bioluminescence images obtained after drug treatment (17 and 24 days) showed a significant reduction of leukemia burden in response to MI-238 alone, while, the greater suppression of leukemia progression was seen in the combination treatment compared with MI-238 or venetoclax alone (Fig. 6B).

Meanwhile, the percentage of Molm13 cells in the murine peripheral blood was quantified by flow cytometry using anti-human CD45 (hCD45) and anti-hCD33 monoclonal antibodies, since hCD45/hCD33 double positive was recognized as the human AML marker (Ehninger et al. 2014). As shown in Fig. 6C and D, MI-238 alone treatment significantly reduced the hCD45^+^ /hCD33 ^+^ cells in the peripheral blood compared with vehicle-treated mice (11.7 ± 3.4% vs. 24.7 ± 3.9%). Although, venetoclax alone also decreased percentage of hCD45^+^ /hCD33^+^ cells (10.5 ± 2.1%), venetoclax in combination with MI-238 could decrease hCD45^+^/hCD33^+^ leukemia cells to 3.0 ± 1.7% (Fig. 6C, D). Similarly, immunohistochemical (IHC) analysis of hCD45^+^ cells in bone marrow also proved that MI-238 treatment alone could significantly decrease tumor burden, while MI-238 in the combination with venetoclax induced a greater reduction in the tumor burden (Fig. 6E, F). In addition, survival analysis revealed that MI-238 alone or in combination with venetoclax could significantly prolong the survival of tumor-bearing mice (vehicle treated mice = 24.6 days, vs MI-238 treated mice = 28 days, vs venetoclax treated mice = 29.8 days, vs combination treated mice = 35.8 days) (Fig. 6G).

### MI-238 treatment alone or in combination with venetoclax is effective in AML patient samples

**Fig. 7:**
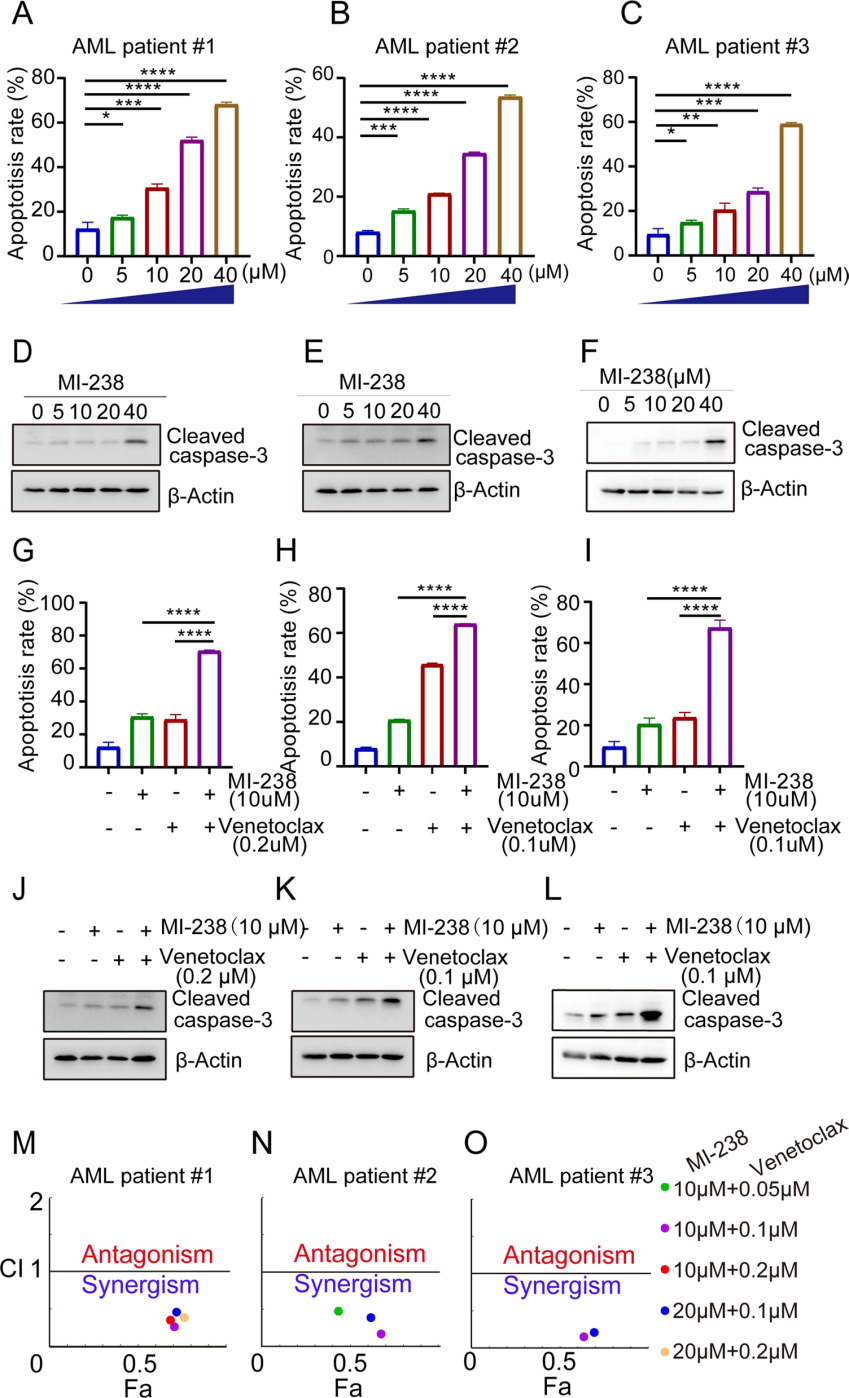
MI-238 treatment alone or in combination with venetoclax effectively induces apoptosis in primary patient AML cells. Bone-marrow mononuclear cells from three different AML patients were treated as indicated for 48 h, and the apoptosis were measured by annexin V staining (**A**–**C**; **G**–**I**) or western blot analysis of caspase 3 cleavage (**D**–**F**; **J**–**L**). The combination index (CI) was calculated by CompuSyn software (**M**–**O**). Data represent mean ± SD. *P < 0.05,  **P < 0.01, ***P < 0.001, and ****P < 0.0001

In order to further validate the therapeutic efficacy of MI-238 and its combination with venetoclax, primary patient AML cells were analyzed. Mononuclear bone marrow cells from 3 different AML patients were treated with increasing concentrations of MI-238 and the apoptosis was analyzed by annexin V staining. Consistent with AML cell line, MI-238 treatment induced apoptosis in AML patient samples in a dose-dependent manner and more than half of bone-marrow mononuclear cells underwent apoptosis in presence of 40 μM MI-238 treatment in all three patient samples (Fig. 7A–C, Additional file 1: Fig. S6). Similarly, we detected increasing cleavage of caspase 3 after treatment of MI-238 (Fig. 7D–F), which confirmed that MI-238 potently induced apoptotic cell death in tumor cells from AML patient samples. Meanwhile, 20 μM of MI-238 treatment failed to induce apoptosis in bone-marrow mononuclear cells from healthy donor (Additional file 1: Fig. S7). Then, we treated patient AML cells with MI-238, venetoclax or their combination to test whether MI-238 could sensitize AML patient samples to venetoclax. As shown in Fig. 7G–I, we detected significantly greater apoptosis in patient AML cells treated MI-238 plus venetoclax compared with cells treated MI-238 or venetoclax alone. Besides, greater cleavage of caspase 3 was detected in patients AML cells treated with MI-238 and venetoclax combination, which further demonstrated that MI-238 is effective in primary patient AML cells (Fig. 7J–L). Besides, a significantly synergistic effects of MI-238 and venetoclax on apoptosis induction in primary AML patient samples (Fig. 7M–O). Collectively, these data demonstrated that MI-238 alone or its combination with venetoclax efficiently induces apoptosis in the bone marrow samples of AML patient, further supporting its therapeutic efficacy to treat AML.

## Discussion

Mcl-1 is a Bcl-2 anti-apoptotic family member with unique properties (Chen et al. 2019). Mcl-1 has a short half-life and its expression is regulated by a variety of survival signals (Senichkin et al. 2020). Multiple E3 ligases and deubiquitinases have been identified to control ubiquitination and proteasome mediated degradation of Mcl-1 (Kim et al. 2021; Morgan et al. 2021; Zhang et al. 2018; Zhong et al. 2005). In addition, Mcl-1 is structurally different from other Bcl-2 anti-apoptotic members at the long amino terminus, which contains two PEST domains, rich in proline (P), glutamic acid (E), serine (S) and threonine (E) amino-acid residues (Chen et al. 2018). This PEST domain bears many residues which could be phosphorylated by protein kinase such as GSK-3, and subsequently results in Mcl-1 degradation (Senichkin et al. 2020). Furthermore, the BH3 binding pocket of Mcl-1 is distinct from that of Bcl-2 and Bcl-xL, which restricts the development of high affinity Mcl-1 inhibitor, and the known BH3 mimetics ABT-737 could effectively inhibit Bcl-2/Bcl-xL, but not Mcl-1 (Souers et al. 2013). Meanwhile, like other Bcl-2 anti-apoptotic members, high frequency of Mcl-1 gene amplification has been observed in various human cancers and the elevated Mcl-1 protein level has also been validated in cancer tissues, which underlines the importance of Mcl-1 for cancer cell survival (Senichkin et al. 2019). And, multiple strategies targeting Mcl-1, including small molecule BH3 mimetics (Kotschy et al. 2016) synthetic peptides fit into Mcl-1 BH3-binding groove (Stewart et al. 2010), covalent allosteric inhibition (Akcay et al. 2016), proteolysis targeting chimera (PROTAC) mediated Mcl-1 degradation (Wang et al. 2019), interfering Mcl-1 transcription (Thomas et al. 2013), have been proved to possess promising anti-cancer efficiency. Here, we show a novel small molecule named MI-238, which could effectively inhibit Mcl-1’s anti-apoptotic function in vitro and selectively induce apoptosis in Mcl-1 proficient cells. The discovery of MI-238 provides a novel drug candidate to target Mcl-1 for future cancer treatment.

In our present study, we confirmed that MI-238 has promising therapeutic efficacy in AML cells, animal model and patients’ samples. And we did not observe adverse effects in mice after administration of MI-238(70 mg/kg), however, pharmacokinetics and safety of MI-238 needed to be further evaluated. Building a series of point of mutations within the BH3 domain of Mcl-1 and measuring the binding affinity of MI-238 to these mutated Mcl-1 proteins would help to identify the exact binding site of MI-238. In light of the importance of Mcl-1 in cancer cell survival, developing Mcl-1 inhibitors have been extensively studied and a number of Mcl-1 inhibitors have been developed (Ramsey et al. 2018; Kotschy et al. 2016; Wang et al. 2019). Recently, the phase I clinical results of Mcl-1 inhibitor AMG-176 in 26 patients with relapsed multiple myeloma (MM) were disclosed. Major side effects including neutropenia, anemia, nausea and diarrhea were observed (Roberts et al. 2021). Meanwhile, significant cardiac side effects were seen in another Mcl-1 inhibitor AMG-397 trial (Carter et al. 2020). Since, Mcl-1 is implicated in normal cardiac myocyte functions (Wang et al. 2013), these cardiac adverse effects probably result from the on-target activity of these compounds and this on-target related side effect may limit therapeutic window of these Mcl-1 inhibitors. Besides the reported Mcl-1 inhibitors, MI-238 in our present study could not induce apoptosis in Mcl-1 deficient cells, demonstrating its high specificity. Nevertheless, further studies needed to be conducted to test whether MI-238 possesses a favorable safety profile and clinical efficacy.

Although Bcl-2 inhibitor venetoclax is highly effective in hematologic malignancies, especially in CLL, the acquired and intrinsic resistance still cause treatment failure (Ramsey et al. 2018). Mcl-1 is considered as the primary venetoclax-resistant factor and inhibition of Mcl-1 could reverse venetoclax resistance in various hematologic cancers (Ramsey et al. 2018). In our present study, we validated that combined treatment of MI-238 and venetoclax exhibited synergistic anti-cancer efficacy in AML cell line, animal model and patients’ samples. However, the mouse model was established with AML cell line. Further validation of the effect of MI-238 with patient-derived xenograft model would provide evidence to support the combination of Mcl-1 inhibitor with venetoclax to treat AML. In summary, our results confirmed that Mcl-1 inhibition is the primary strategy to overcome venetoclax resistance and our study provided a novel leading compound which could be utilized as venetoclax sensitizer.

## Conclusions

In summary, our study provides a novel and selective Mcl-1 inhibitor, MI-238, which specifically induces apoptosis in Mcl-1 proficient cells, but not in Mcl-1 deficient cells. MI-238 treatment alone or its combination with venetoclax effectively kills AML cells in vitro, AML mouse model and primary AML patient samples.

## Supplementary Information

**Additional file 1: Fig. S1.** MI-238 selectively suppressed cell growth of H1299 parental cells but not Mcl-1 knockout (KO) cells. **Fig. S2.** H1299 Mcl-1 KO cells or mouse embryonic fibroblast (MEF) cells do not depend on Mcl-1 for survival. **Fig. S3.** Primary bone marrow cells with elevated expression level of Mcl-1 is more sensitive to MI-238. **Fig. S4.** The profiles of apoptosis analysis by annexin V/PI staining in Molm13 cells treated with indicated concentrations of MI-238 and venetoclax or their combinations. **Fig. S5.** The activation of Bak in Molm13 cells after 24 h of indicated treatment was analyzed by flow cytometry. **Fig. S6.** The representative annexin V/PI staining profiles of primary patient AML cells treated with indicated concentrations of MI-238 for 48 h. **Fig. S7.** 20 μM of MI-238 failed to induce apoptosis in mononuclear bone marrow cells from healthy donor.

## Data Availability

The data during the current study are available from the corresponding author on reasonable request.

## Abbreviations

AML: Acute myeloid leukemia
CLL: Chronic lymphocytic leukemia
FP: Fluorescence polarization
MEF: Mouse embryonic fibroblast
KO: Knockout
IP: Immunoprecipitation
CI: Combination index
MM: Multiple myeloma
IHC: Immunohistochemistry
