# Supplementary material for: A novel Mcl-1 inhibitor synergizes with venetoclax to induce apoptosis in cancer cells
Source: Mol Med. 2023 Jan 19;29:10. doi: 10.1186/s10020-022-00565-7 (PMC9854187; doi:10.1186/s10020-022-00565-7)
Supplement: Supplementary file 1 — Additional file 1: Fig. S1. MI-238 selectively suppressed cell growth of H1299 parental cells but not Mcl-1 knockout (KO) cells. Fig. S2. H1299 Mcl-1 KO cells or mouse embryonic fibroblast (MEF) cells do not depend on Mcl-1 for survival. Fig. S3. Primary bone marrow cells with elevated expression level of Mcl-1 is more sensitive to MI-238. Fig. S4. The profiles of apoptosis analysis by annexin V/PI staining in Molm13 cells treated with indicated concentrations of MI-238 and venetoclax or their combinations. Fig. S5. The activation of Bak in Molm13 cells after 24 h of indicated treatment was analyzed by flow cytometry. Fig. S6. The representative annexin V/PI staining profiles of primary patient AML cells treated with indicated concentrations of MI-238 for 48 h. Fig. S7. 20 μM of MI-238 failed to induce apoptosis in mononuclear bone marrow cells from healthy donor. [file 10020_2022_565_MOESM1_ESM.docx]

Supplementary Data to

**A novel Mcl-1 inhibitor synergizes with venetoclax to induce apoptosis in cancer cells**

Tianming Zhao^1†^, Qiang He^2†^, Shurong Xie^1†^, Huien Zhan^1†^, Cheng Jiang^3^, Shengbin Lin^2^, Fangshu Liu^1^, Cong Wang^4^, Guo Chen^2,4^*, Hui Zeng^1^*

^1^Department of Hematology, The First Affiliated Hospital of Jinan University, Guangzhou, 510630, China.

^2^Department of Medical Biochemistry and Molecular Biology, School of Medicine, Jinan University, Guangzhou, 510632, China.

^3^Jiang Su Key Laboratory of Drug Design and Optimization, Department of Medicinal Chemistry, China Pharmaceutical University, Nanjing, 210009, China.

^4^School of Biopharmacy, China Pharmaceutical University, Nanjing, 211198, China.

†Tianming Zhao, Qiang He, Shurong Xie and Huien Zhan contributed equally to this work.

*Correspondence: Dr. Hui Zeng, E-mail: [androps2011@hotmail.com](mailto:androps2011@hotmail.com). ORCID: 0000-0002-4498-8888. Department of Hematology, The First Affiliated Hospital of Jinan University, Guangzhou, 510630, China. Tel:020-38688696. Dr. Guo Chen, E-mail: [gchen84@jnu.edu.cn](mailto:gchen84@jnu.edu.cn). ORCID: 0000-0001-8886-1873. Department of Medical Biochemistry and Molecular Biology, School of Medicine, Jinan University, Guangzhou, 510632, China; School of Biopharmacy, China Pharmaceutical University, Nanjing, 211198, China.

**Supplementary methods**

**Cell viability:**

The anti-tumor efficacy of MI-238 against AML cells was determined by Cell Counting Kit-8 (CCK-8, Dojindo, Japan). AML cells were cultured in 1640 or IMDM medium (Biological Industries, USA) supplied with 5% fetal bovine serum (Biological Industries, USA) at 37°C in an atmosphere containing 5% carbon dioxide. For CCK-8 assay, 100μl medium containing 2 × 10^4^ cells were seeded into 96-well culture plates and treated with MI-238 at different concentrations. Vehicle control was treated with DMSO. After 48 h treatment, 10 μl of CCK-8 solution was added to each well, and the incubation continued for 2 h at 37°C. Absorbance was read at 450 nm using a Varioskan LUX. The IC_50_ of the compound was calculated with GraphPad Prism software 8.0 (GraphPad, San Diego, CA, USA).

**Calculation of combination index:**

The combination index (CI) values were analyzed using CompuSyn software, which has been used extensively to evaluate the compound-induced cytotoxicity synergy. CI value > 1 are considered to be antagonistic effects and CI value < 1 indicates synergistic effect. Strength of synergism can be defined as follows: CI value < 0.1 very strong synergism, CI value = 0.1-0.3 strong synergism; CI value = 0.3-0.7 moderate synergism; CI value = 0.7-0.9 slight synergy; CI value = 0.9–1.0 purely additive effect.

**Cell fractionation:**

Cytoplasmic and mitochondrial proteins were extracted to detect the localization of cytochrome C using Cytoplasmic and Mitochondrial Protein Extraction Kit (Sangon Biotech, Shanghai, China) according to the manufacture’s protocol. Briefly, 2×10^7^ AML cells were collected and washed twice with cold PBS. Samples were resuspended with protein extraction buffer supplemented with protease inhibitor, phosphatase inhibitor and DTT, and homogenized for 30-50 times on ice. The resulting cell lysates were centrifuged for 30 minutes at 12000rpm. The pellet represents the mitochondria and the supernatant contains cytoplasmic proteins.The above two fractions were used for Western blot to detect cytochrome C.

**Fluorescence polarization (FP) assay:**

Mcl-1 recombinant protein (50 nM) was incubated with 5 nM of fluorescent Bak BH3 peptide (TAMRA-GQVGRQLAIIGDDINR) in the absence or presence of increasing concentrations of MI-238 in the binding buffer [50 mM Tris (pH 8.0), 150 mmol/L NaCl, 0.1% bovine serum albumin (BSA), and 5 mM DTT] in black 1,536-well microplates. Plates were incubated at room temperature for 1h and FP values (in millipolarization units) were measured using Envision multi-label plate reader (Perkin Elmer). The excitation filter was at 540 ± 20 nM and emission filter at 590 ± 20 nM. Data analysis and inhibitory constant (Ki) value were determined by GraphPad prism software as described.

**Supplementary figures**

**
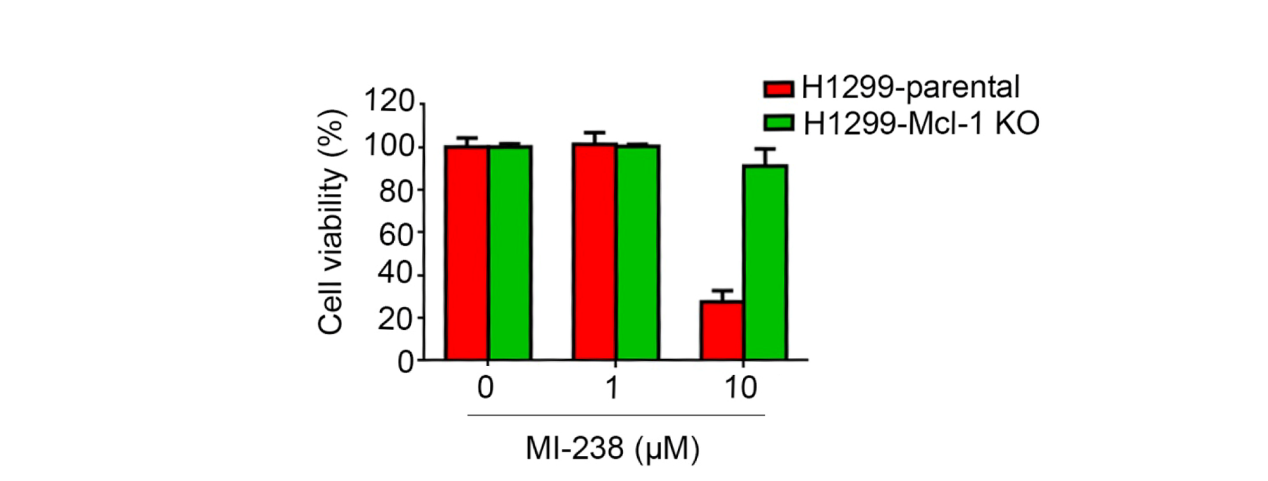
**

**Figure S1.** MI-238 selectively suppressed cell growth of H1299 parental cells but not Mcl-1 knockout (KO) cells. 5×10^3^ of H1299 parental cells or Mcl-1 KO cells were seeding into 96 well-plate and grew in presence of indicated concentrations of MI-238 for 72 hours and the cell viability were measured by CCK-8.


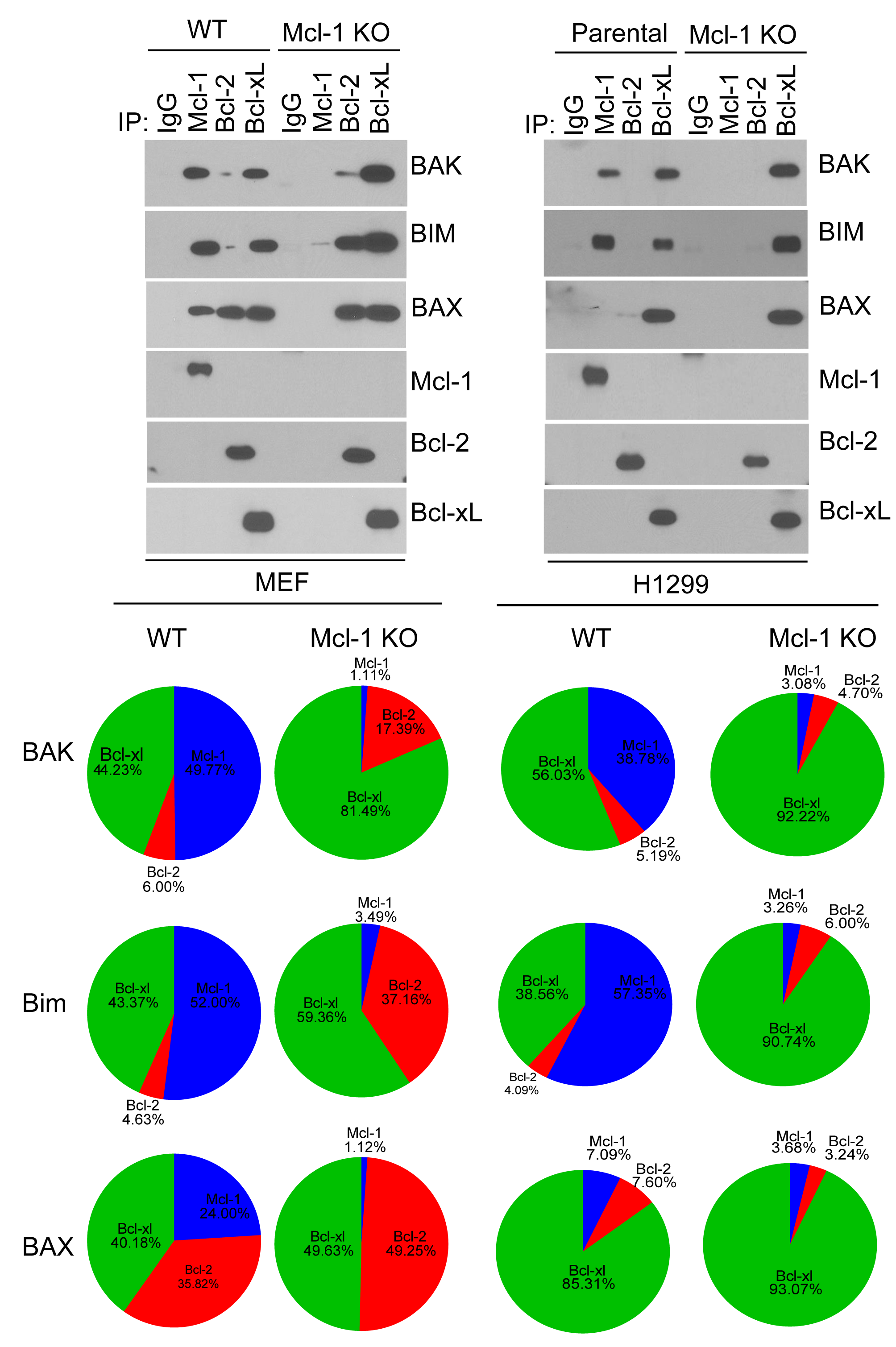


**Figure S2.** H1299 Mcl-1 KO cells or mouse embryonic fibroblast (MEF) cells do not depend on Mcl-1 for survival. (A) Cell lysates from H1299 (parental vs. Mcl-1 KO) or MEF (WT vs. Mcl-1 KO) cells were subjected to immunoprecipitation (IP) assay using anti-Mcl-1, anti-Bcl-2 or anti-Bcl-xL antibodies, and the bound BH3-only proteins including Bax, Bak and Bim were analyzed by western blot. (B) The percentage of Bak, Bim and Bax association with Mcl-1, Bcl-2 and Bcl-xL in Mcl-1 parental or KO cells were quantified.


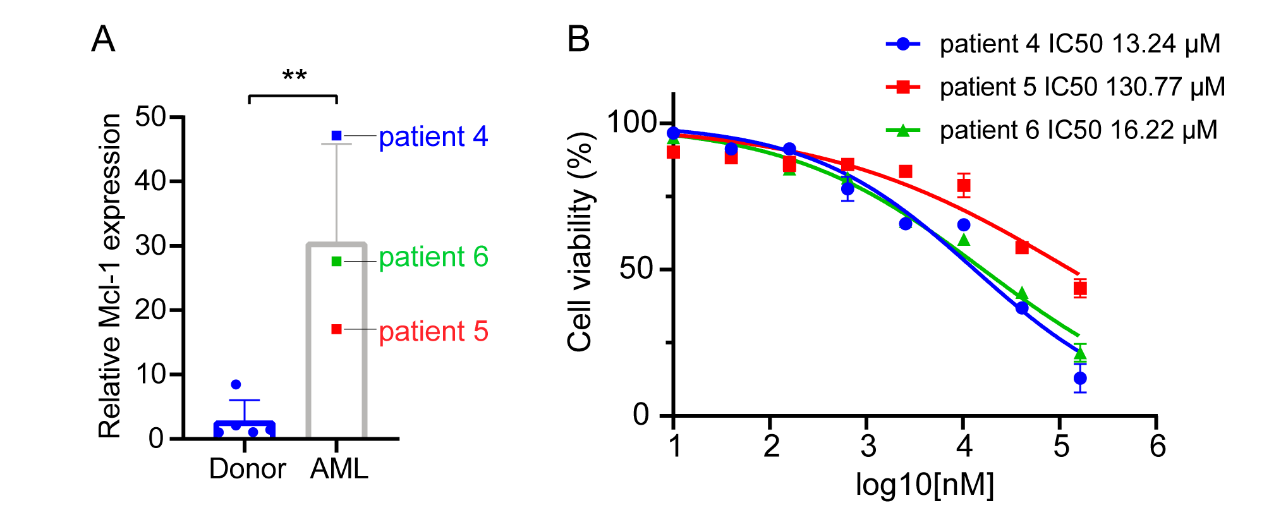


**Figure S3.** Primary bone marrow cells with elevated expression level of Mcl-1 is more sensitive to MI-238. (A) Mcl-1 expression levels were measured in healthy donors and patients with AML by qPCR. (B) The cell viability of 3 AML patients in the presence of increasing concentrations of MI-238. Cell viability was analyzed by cell counting kit-8 (CCK8) assay and the IC50 were determined.


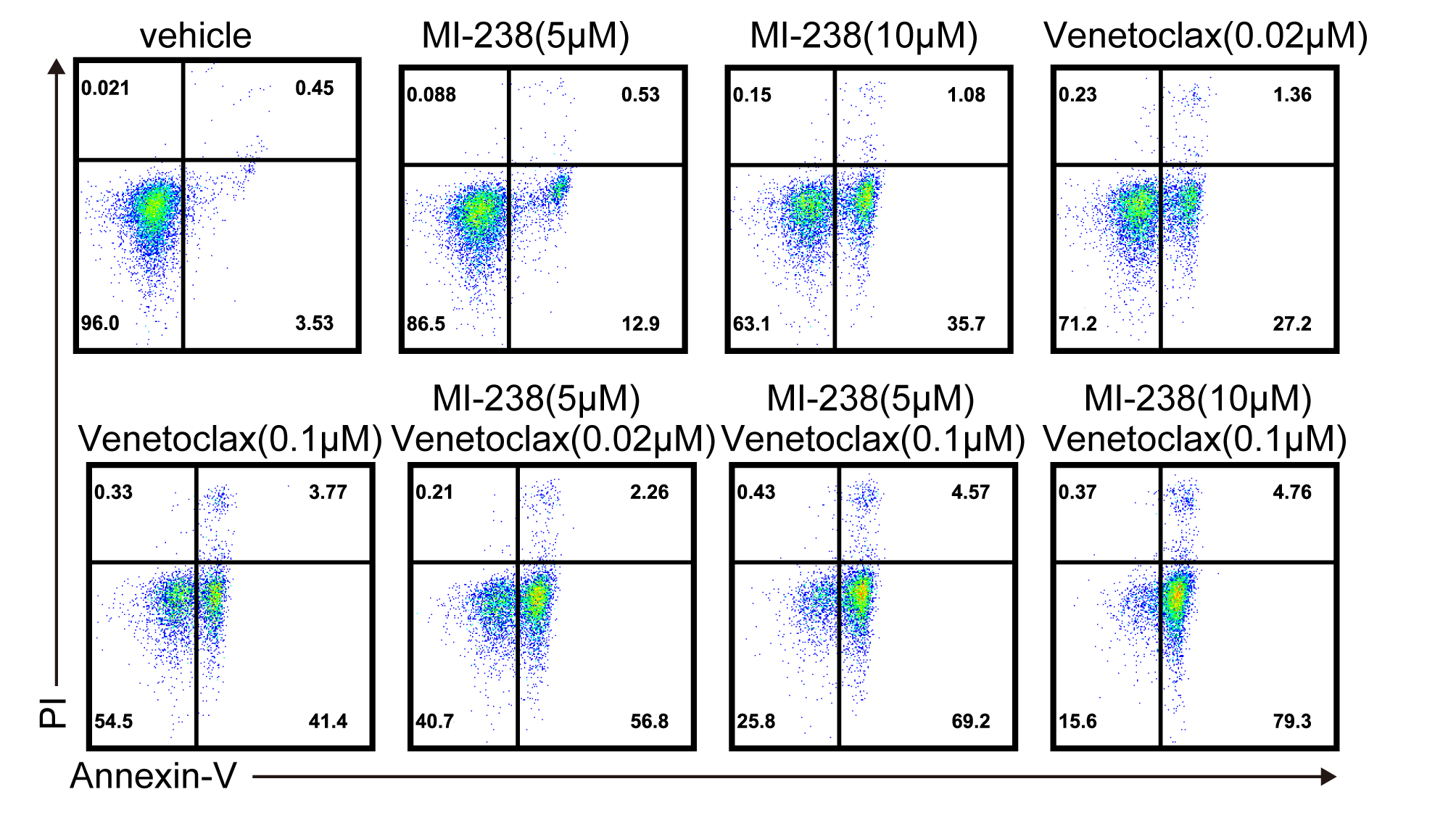


**Figure S4.** The profiles of apoptosis analysis by Annexin V/PI staining in Molm13 cells treated with indicated concentrations of MI-238 and venetoclax or their combinations.


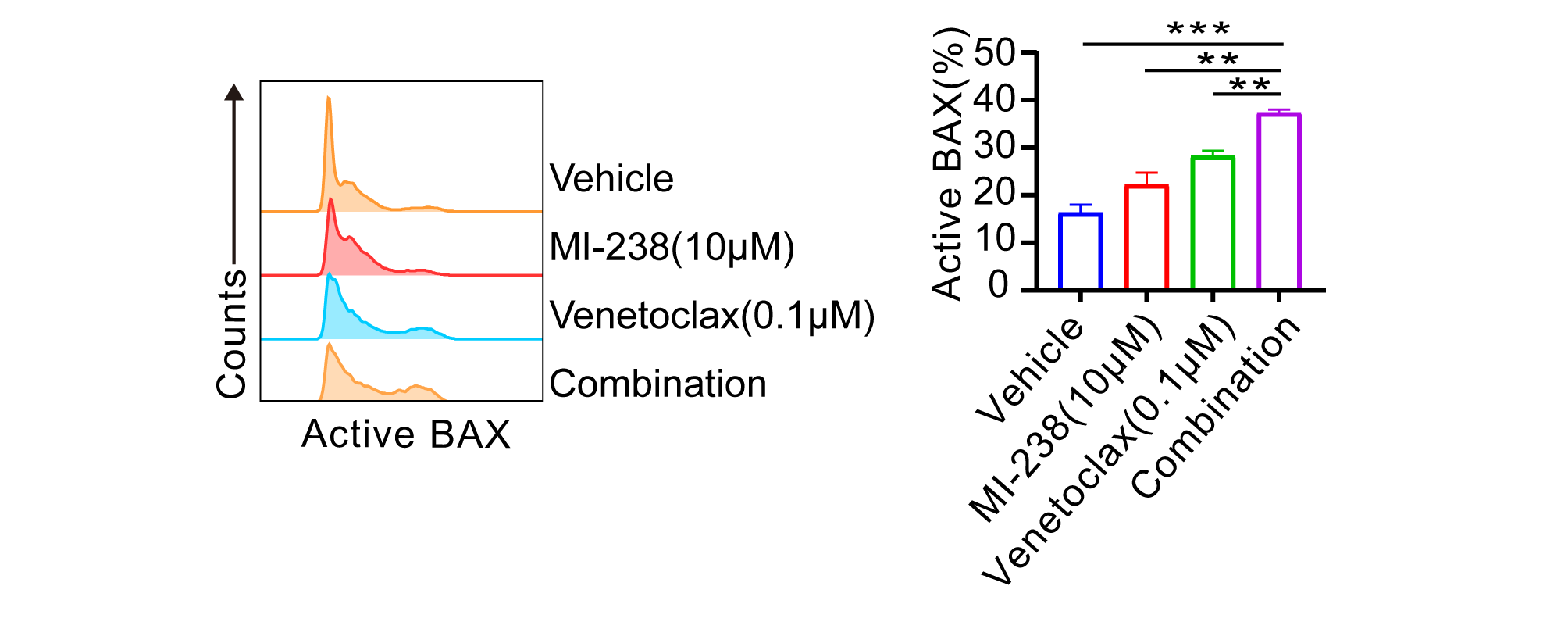


**Figure S5.** The activation of Bak in Molm13 cells after 24 hours of indicated treatment was analyzed by flow cytometry.


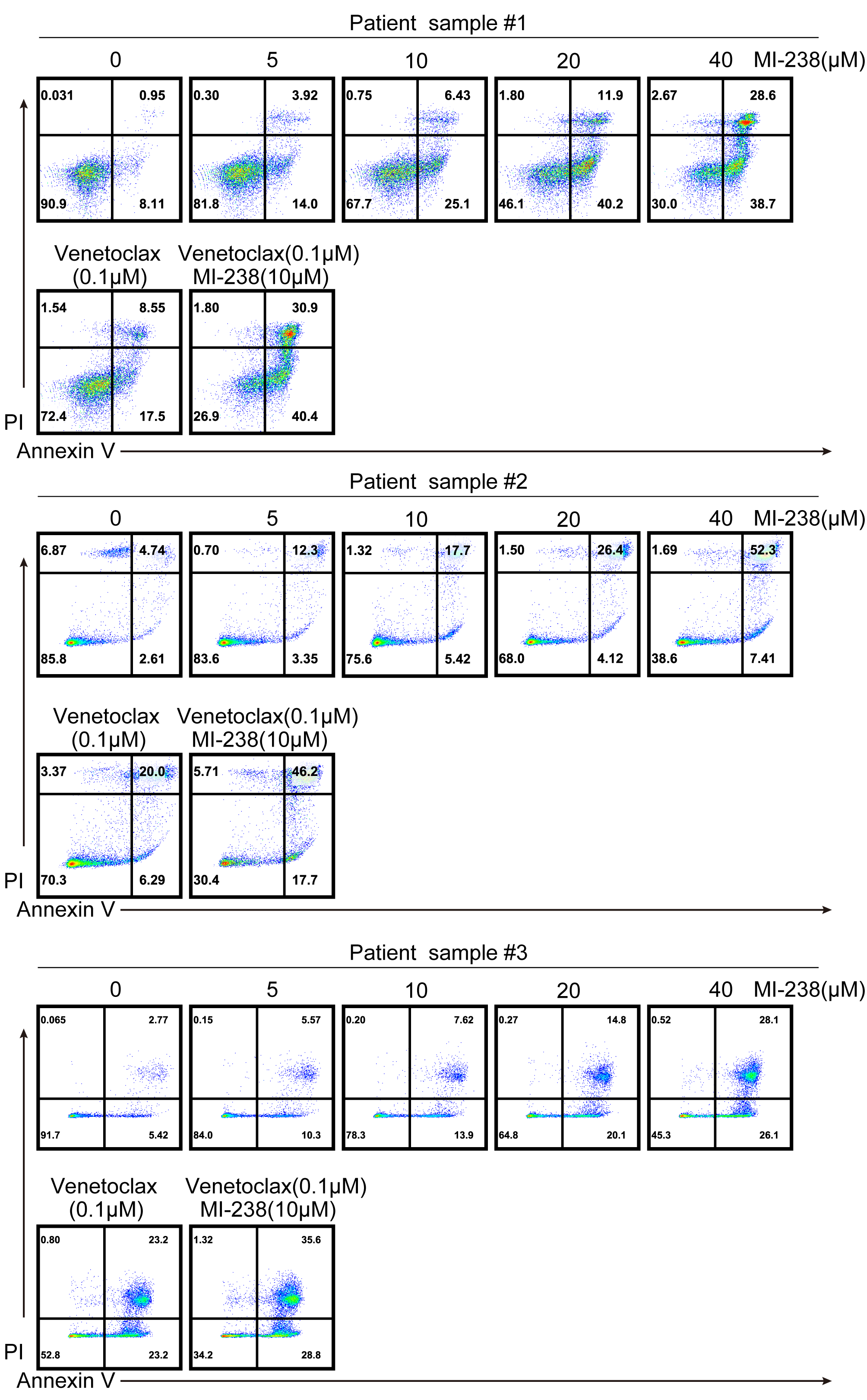


**Figure S6.** The representative Annexin V/PI staining profiles of primary patient AML cells treated with indicated concentrations of MI-238 for 48 hours.


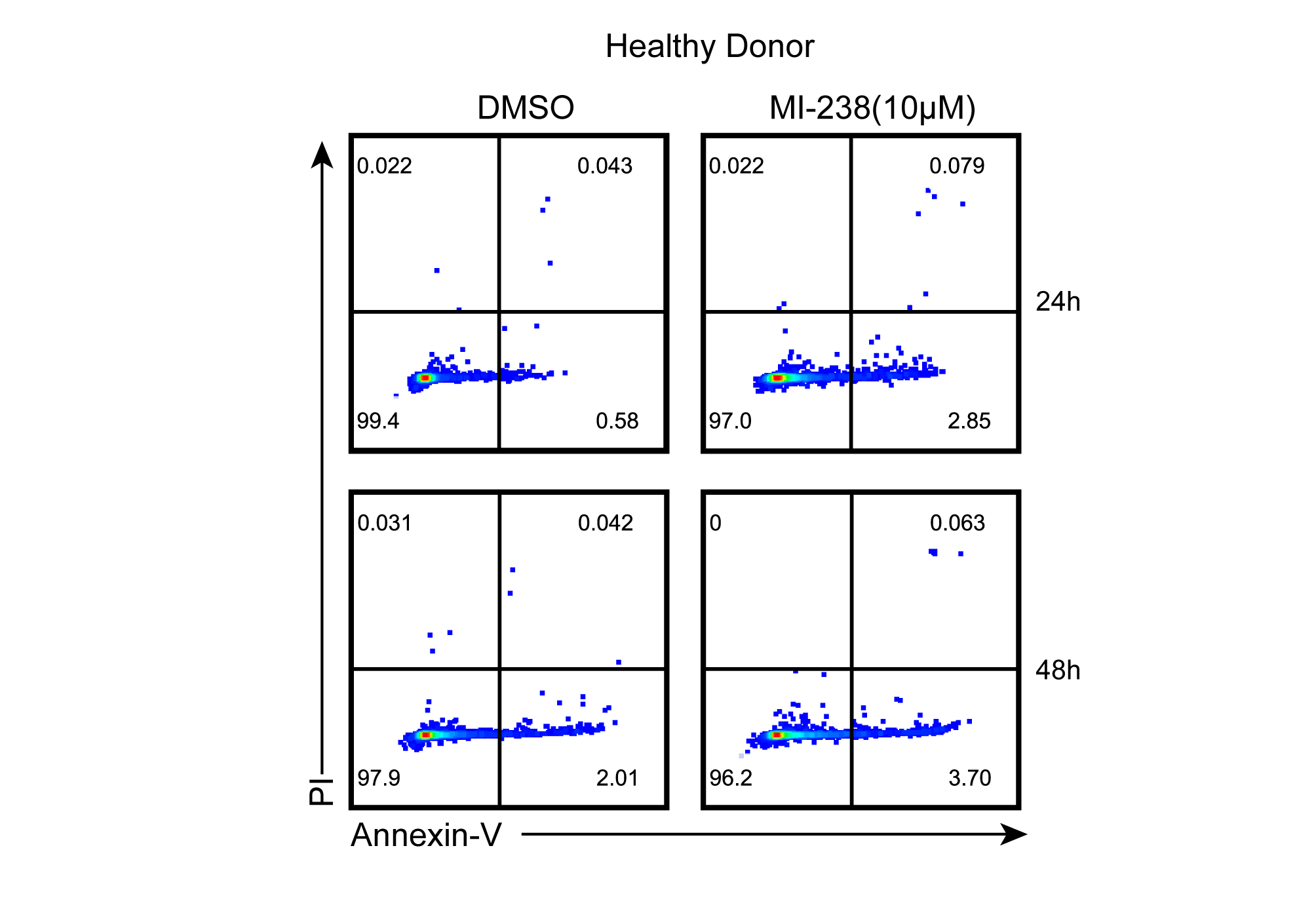


**Figure S7.** 20 μM of MI-238 failed to induce apoptosis in mononuclear bone marrow cells from healthy donor.
